# Soundscape Preference of Urban Residents in China in the Post-pandemic Era

**DOI:** 10.3389/fpsyg.2021.750421

**Published:** 2021-12-22

**Authors:** Jinxuan Liu, Jian Xu, Zhicai Wu, Yuru Cheng, Yuxin Gou, Jesse Ridolfo

**Affiliations:** ^1^Department of Tourism Management, South China University of Technology, Guangzhou, China; ^2^State Key Laboratory of Subtropical Building Science, Guangzhou, China; ^3^Guangdong Tourism Strategy and Policy Research Center, South China University of Technology, Guangzhou, China; ^4^Xi’an Liangjiatan International School, Xi’an, China

**Keywords:** COVID-19, soundscape preference, survey, China urban, public landscape, post-pandemic era

## Abstract

This research aims to explore the reality of the soundscape preferences of Chinese urban residents in general public landscape in the post-pandemic era, and then to propose design recommendations to meet the practical needs of people’s preferences for landscape—especially soundscapes—in the post-pandemic era. In this study, we utilized the subjective evaluation method to conduct an online questionnaire in 29 Chinese provinces which experienced severe pandemic caseloads and collected 860 valid responses. This study revealed people’s preference for landscape and soundscape in the post-pandemic era. We further studied the correlation between landscape preference and soundscape preference, analyzed the influence of living conditions on soundscape preference, founded the effects of personal characteristics and living conditions on soundscape preference, and explored the strongest influence factors on soundscape preference through the establishment of automatic linear model. The results revealed a positive correlation between life happiness and soundscape preference, whereas wearing masks significantly reduced soundscape perception ratings and people who have been vaccinated are more tolerant of various noises. Moreover, based on these analysis results, the design recommendations on landscape (overall landscape, plant, and tour space), soundscape construction of caring for vulnerable groups (teenagers and children, elderly people, and disabled and unhealthy) has been discussed.

## Introduction

The novel coronavirus pneumonia (COVID-19) is the most impactful and deadly global public health event in the world in the past 100 years (Xu, 2020). Many countries around the world have chosen lockdown and restrictions on people’s mobility (home and community isolation) as the main strategies to combat the COVID-19 pandemic. These policies have increased residents’ psychological anxiety and have caused harm to their physical and mental health (Liu et al., 2020). These actions have significantly modified urban soundscape, opening up an unprecedented opportunity for research in soundscape field (Asensio et al., 2020). Under the influence of pandemic, urban residents’ desire for tourism, fitness, and leisure has increased, and residents have shown a strong physical and mental demand for urban public landscape (Wang et al., 2020; Lenzi et al., 2021), meanwhile, urban public landscape is facing substantial challenges as a result of the measures taken to limit the spread of the virus (Fu et al., 2020). It is a topic for reflection whether and what changes have taken place in people’s soundspace preference in the urban public landscape in the post-pandemic era (Chen and Bai, 2021). In the pandemic and post-pandemic era, the change in the soundscape preference in general landscape space has become an essential factor to consider when designing public space. The preference for landscape and soundscape will continue to evolve as the pandemic continues and actions are taken to prevent its spread. This article aims to explore the reality of the soundscape preferences of Chinese urban residents in general landscape spaces under the influence of COVID-19 and then to propose design recommendations to meet the practical needs of people’s preferences for landscape—especially soundscapes—in the post-pandemic era.

At present, the pandemic has put forward new requirements for the landscape and soundscape design of urban public spaces. Urban public space refers to the open space between architectural entities in a city or urban environment, mainly contains natural environments, such as mountains, forests, water systems, as well as artificial parks and green spaces, it is used for urban residents to conduct public communication and hold various life and social activities (Carr et al., 1993; Xi, 2021). At present, few method based on soundscape design was adopted for visual-centered tendency in landscape plan (Lian et al., 2020), but a large number of studies have shown that soundscape has a critical impact on people’s viewing experience (Zhang, 2014; Kogan et al., 2021). Soundscape design attempts to discover principles and to develop techniques by which the social, psychological, and esthetic quality of the soundscape may be improved (Alfredo et al., 2014; Kogan et al., 2021). Soundscape (acoustic environment perceived and understood by an individual or society) can impact human health (Aletta et al., 2018a), and an upbeat soundscape is often associated with faster stress recovery and better self-reported health status (Kang, 2006; ISO, 2014; Aletta et al., 2018b; Liu et al., 2019a). Studies have found that natural sound can reduce the human heart rate (HR), respiratory frequency (RF), and respiratory depth (RD) (Li and Kang, 2019), natural sounds—such as bird songs—can reduce anxiety, relieve stress, and promote emotional stability (Annerstedt et al., 2013). Acoustics also has a significant impact in the medical and health field (Baird and Samson, 2015). Therefore, pleasant sounds can enhance people’s viewing experience in an urban environment (Aletta et al., 2018a,b; Liu et al., 2019a). So far, many scholars have conducted detailed studies on cities (Zhang, 2016), villages (Ren, 2016), airports (Kitapci and Galbrun, 2019), and commercial spaces (Song et al., 2011; Meng and Kang, 2018), urban pedestrian streets (Yu et al., 2014) and historical districts (Zhou et al., 2012), etc. Although emerging studies have discussed the potential benefits of soundscape in mental restoration, few have investigated how soundscape renews and re-energizes people, especially in the face of the current public challenge of the COVID-19 crisis. In view of the pandemic, a moderated mediation model to reveal that natural soundscapes have great restorative benefits for visitors (Qiu and Zhang, 2021); a set of descriptors is outlined which better enables the application of more novel approaches to the evaluation of the effect of this new soundscape on people’s subjective perception under the pandemic (Asensio et al., 2020). A case study of the human perception of environmental sounds in an urban neighborhood, discussed how such changes in the acoustic environment of the site under the pandemic (Lenzi et al., 2021). An online survey administered to 464 home workers in January 2021 in London, utilized a previously developed model for the assessment of indoor soundscapes to describe the affective responses to the acoustic environments (Torresin et al., 2021). These new literature make many ground-breaking contributions to the soundscape of the pandemic, and these results influence the further exploration of urban public space soundscape and the consideration of questionnaire questions in this study. However, the research on soundscape preferences in urban public landscape in the pandemic and post-pandemic era requires additional exploration. Under the demand of both pandemic prevention and leisure and fitness, exploring the residents’ preference for urban general landscape and soundscape can guide the urban public landscape design in the post-pandemic era.

To achieve this result, this study used the subjective evaluation method to conduct an online questionnaire in 29 Chinese provinces with a high incidence of COVID-19 cases, from which 860 valid questionnaires were collected. This study revealed people’s preference for landscape and soundscape after the pandemic, studied the correlation between landscape and soundscape preference, analyzed the influence of living conditions on soundscape preference, founded the effects of personal characteristics and living conditions on soundscape preference, and explored the strongest influence factors on soundscape preference through the establishment of automatic linear model. The main findings of the study revealed the direct impact of epidemic prevention measures on soundscape evaluation, summarized the main influencing factors of soundscape preference in the post-pandemic era, found the impact of individual factors on soundscape evaluation under the pandemic influence. Moreover, based on these analysis results, the design recommendations on landscape (overall landscape, plant, and tour space), soundscape construction of caring for vulnerable groups (teenagers and children, elderly people, and disabled and unhealthy) has been discussed.

## Methodology

**FIGURE 1:**
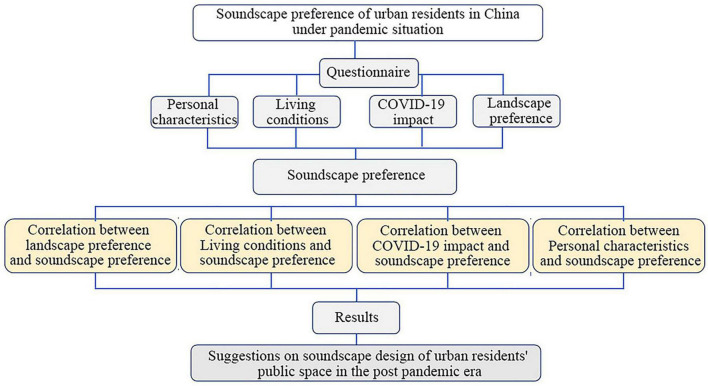
Research concepts.

This study adopts theoretical research-question, investigation-data, analysis-proposed, and strategy research-design recommendations (Figure 1). Based on the concept and academic background of urban public space soundscape preferences, a questionnaire surveyed urban residents within 29 Chinese provinces with a high pandemic incidence to study the soundscape preferences of general landscape spaces in the post-pandemic era.

### Research Area

**FIGURE 2:**
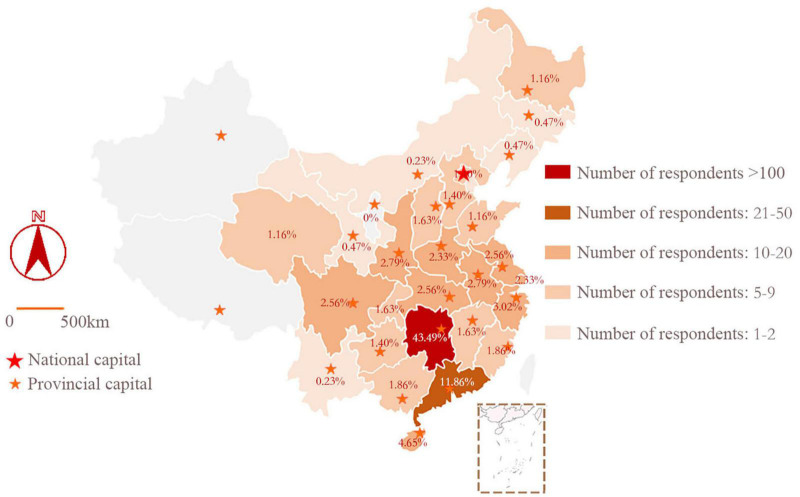
Survey area and quantity distribution.

To ensure the comprehensiveness of the questionnaire, the study mainly selected Hunan Province (29.81%, 256 responses), Guangdong Province (11.86%, 102 responses), Hubei Province (8.56%, 74 replies), Hainan Province (7.65%, 66 responses), Zhejiang Province (5.02%, 43responses), Hebei Province (4.40%, 38 responses), and an additional 23 provincial most severely infected administrative regions. Among them, Hubei Province is the most seriously affected by the pandemic. In total, 860 valid questionnaires were collected. The distribution and proportion of questionnaires in each province are shown in Figure 2.

### Survey Object and Questionnaire Design

**FIGURE 3:**
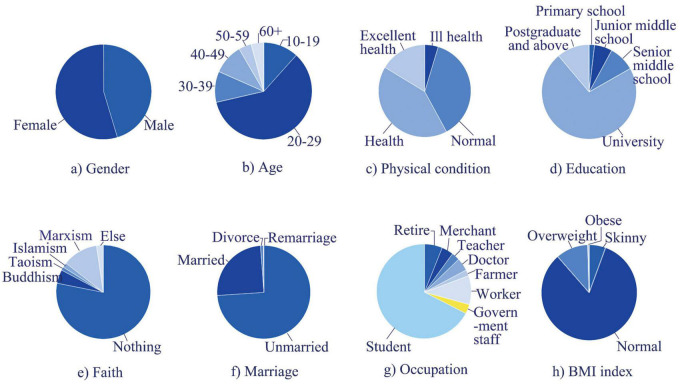
Distribution of essential characteristics of respondents. BMI is a commonly used index to measure the degree of obesity and health in the world. The average value of BMI is between 20 and 25, more than 25 is overweight, and more than 30 is obesity. **(a)** Gender. **(b)** Age. **(c)** Physical condition. **(d)** Education. **(e)** Faith. **(f)** Marriage. **(g)** Occupation. **(h)** BMI index.

To ensure scientific rigor, this study uses questionnaires to conduct soundscape perception research (Ren et al., 2012, 2015; Meng and Kang, 2018; Pickens et al., 2019). This method is more suitable for research on the subjective evaluation of a population. We conduct a random sampling survey of people aged 10–60 who (have normal sound hearing) can subjectively evaluate public landscape and soundscapes in selecting survey subjects. The proportion of the essential characteristics of the respondents is shown in Figure 3.

The survey was conducted during a relatively stable time in the pandemic and post-pandemic era within China, from January to February 2021. This period is the Chinese New Year holiday; as such, people have more free time for exercise, leisure activities, and stress reduction than usual.

The research uses “Questionnaire Star” as the research tool. “Questionnaire Star” is a professional online questionnaire platform in China, which has been used to conduct more than 120 million questionnaires. We use random sampling to generate a QR code and distribute the questionnaire through WeChat.

This study summarized literature to produce 65 questions to survey the overall soundscape preferences of the respondents on three parts: the basic situation of the respondent (question 1-18), the landscape preference and overall feeling (question 19–30), and the soundscape preferences (question 35–64). The basic information of the respondents includes gender, age, occupation, annual family income, personal health, whether they have been infected with the coronavirus, and whether they have been vaccinated against the coronavirus. Questions about the surrounding greening environment were designed to investigate the greening rate, distance to the nearest green space, and the frequency of public landscape space use in the post-pandemic era. After the pandemic, the general landscape space preference includes several common public landscape space types, such as business districts, streets, squares, grasslands, forests, rivers, seashores, and fields (Guo, 2010). The comprehensive questionnaire is mainly designed to evaluate the preference of the soundscape. According to the ecological and semantic nature of the soundscape, the evaluation divides the sounds into six categories: traffic sounds, mechanical sounds, human activity sound, natural sound, livestock sound, and melody (Brown et al., 2011). Moreover, the Likert five-level scale was used to give evaluation levels for the soundscape: −2: immensely dislike, −1: dislike, 0: no preference, 1: like, and 2: immensely like (Zube, 1984). In addition, in order to avoid order effect, the second group (question 19–30) and the third group (question 35–46), When the respondents answer the questions in the “Questionnaire Star” mobile applet, the questions order will be randomly selected in group. Moreover, for the third group (question 35–46), half of the survey questions option is random adjustment has become from “2: immensely like, 1: like, 0: no preference, −1: dislike, and −2: immensely dislike”, while the other half the survey questions option order are “−2: immensely dislike, −1: dislike, 0: no preference, 1: like, and 2: immensely like”.

Appear with each question, audio is played (35 groups of sounds, 3 s each) to ensure that the respondent receives the same sound when making an evaluation (Ren et al., 2015); Before the questionnaire was distributed, 20 people were tested, the total average time to fill in the questionnaire is about 5 min (5 s to answer a question on average), it is considered reasonable to answer the questionnaire for no more than 10 min (Meng and Kang, 2018); a progress bar is provided to check the progress of the questionnaire. Furthermore, there is a lottery at the end of the questionnaire designed to increase the enthusiasm for participating and sharing. To remove invalid questionnaires and improve the survey’s reliability, two questions are designed (e.g., please select 0) to eliminate weak responses (Ren et al., 2012).

### Reliability and Validity Analysis of Questionnaire

The reliability and validity of the questionnaire data were tested using SPSS26.0 software. The research uses Cronbach α to analyze the reliability of the questionnaire. Literature suggests that if the value of Cronbach α is above 0.5 to 0.6, the results are reliable (Lu, 2004). The calculated responses of the questionnaire all meet this reliability standard: traffic machinery (0.835), human activity sound (0.879), natural sound (0.857), livestock sounds (0.889), and music (0.799). The overall perception factor reliability is 0.926. It can be concluded that the reliability of this questionnaire meets the survey requirements. To test the validity of the questionnaire, the study applied factor analysis to test the validity of KMO. The result was 0.907, which satisfies the conditions for factor analysis (KMO ≥ 0.6) (Lu, 2004), indicating that the validity of the questionnaire also meets the requirements. The Bartlett sphere test approximates the chi-square value of 9252.826, which corresponds to a probabilistic value of 0.000 (*P* < 0.01), suggesting that the questionnaire has a significant correlation and that its analytical data is valid.

## Results

### Soundscape Preference Overview in the Post-pandemic Era

As shown in Table 1, the most preferred sounds of urban residents in public landscape spaces in the pandemic and post-pandemic era are the sound of natural flowing water (0.89), wind blowing leaves (0.82), bird songs (0.77); popular music (0.32), heavy rain (0.31), temple bells (0.28), wedding music (0.09) and festival singing and dancing (0.03), indicating that people prefer natural sounds and music in public landscape spaces. The results of previous studies support these findings (Kang and Yang, 2002; Liu et al., 2019b).

**TABLE 1:** Average description of overall soundscape preference of Chinese urban residents in public space under the pandemic situation.

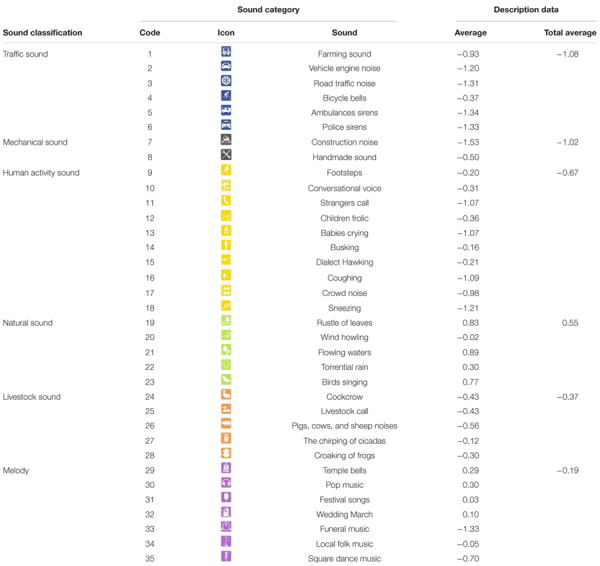

**FIGURE 4:**
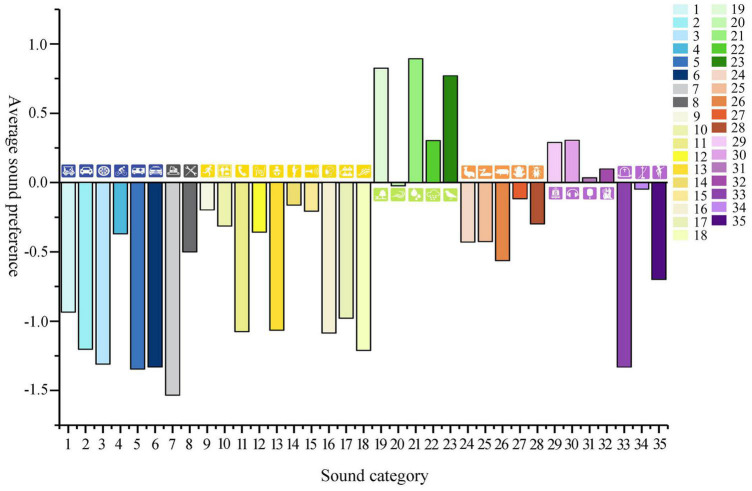
Columnar distribution of the mean value of soundscape preference (code is shown in Table 1).

The sounds that are highly disliked by urban residents include construction noise (–1.54), ambulance sounds (–1.34), funeral music (–1.33), police car sirens (–1.33), and road traffic noise (–1.31). These results are similar to previous conclusions (Zhang et al., 2018). However, it is worth noting that people’s sensitivity to emergencies and deaths has increased under the influence of the pandemic, and the aversion of ambulance sounds, funeral music, and police car sirens is significantly higher than the noise of road traffic. Studies have shown that people’s preferences often do not lie in the sound itself but their positive or negative emotions. Some long-term soundscape memories are preserved, which will produce different soundscape emotions (Liu and Kang, 2016). The conclusion further verified that positive or negative feelings brought about by sounds impact the degree of soundscape preference and that in the pandemic and post-pandemic era, specific sounds can have a particularly negative effect on people’s psychological perception, the specific data is shown in Figure 4.

### Correlation Between Landscape and Soundscape Preference in the Post-pandemic Era

According to the correlation analysis (Tables 2, 3), the type of public landscape space, landscape structural design, and water landscape all affect the subjective evaluation of soundscape. In terms of overall landscape perception, those who prefer commercial areas demonstrate relatively acceptable to the sound of police car sirens. Still, they prefer the sound of children’s frolicking, babies crying, frogs, cicadas, wind blowing leaves, birds, chickens, and square dancing. People who prefer in urban environments have a higher degree of preference for square dances and a relatively lower degree of preference for natural sounds. Those who prefer rural areas—grasslands, woods, mountains, rivers, seaside, and field landscape—show preference for natural sounds, sounds of livestock, children’s frolicking, and farming work have a higher degree of preference.

**TABLE 2:** Correlation between public landscape space preference and soundscape preference under pandemic situation (code is shown in Table 1).

Sound classification		22. Purpose of going to urban park						23. Impact of the pandemic situation				Landscape preference	
		green space under the pandemic situation						on living conditions					
		View	Rest	Play	Recuperate	Motion	Walking	Family	Life	Appreciation	Mask	Sculpture	Waterscape
							pets	Day	state	mood	experience		
Traffic sound	1	−0.027	0.088	0.003	0.013	−0.087	−0.031	0.007	−0.005	0.032	0.080	−0.035	0.080
	2	0.026	0.057	0.021	−0.006	−0.095*	0.023	−0.043	0.087	0.031	0.105*	−0.020	0.030
	3	−0.041	−0.001	−0.020	−0.008	0.000	−0.030	0.017	0.034	0.044	0.097*	−0.012	0.025
	4	0.068	0.062	0.034	0.000	−0.030	0.004	0.005	−0.003	−0.031	0.113*	0.012	0.014
	5	0.024	0.056	−0.004	0.044	0.063	−0.028	−0.010	0.098*	0.019	0.110*	−0.072	−0.069
	6	−0.006	0.084	0.039	0.012	0.076	−0.012	−0.028	0.132**	0.039	0.106*	−0.071	−0.014
Mechanical sound	7	−0.015	0.037	0.011	0.005	−0.035	−0.064	0.026	0.046	0.064	0.090	−0.056	−0.056
	8	0.074	0.086	0.061	0.014	−0.005	0.045	−0.015	−0.070	0.029	0.046	−0.038	−0.040
Human activity sound	9	0.036	0.045	0.050	−0.068	0.085	−0.031	0.043	−0.015	0.008	0.083	0.037	0.058
	10	0.030	0.052	0.034	−0.026	0.030	−0.057	0.075	0.007	−0.010	0.051	0.041	0.021
	11	0.040	0.132**	0.049	0.000	0.002	−0.052	0.038	0.094	0.045	0.088	0.025	−0.016
	12	0.027	0.031	0.047	0.020	0.006	−0.114*	0.145**	0.025	−0.069	0.069	−0.010	0.076
	13	0.054	0.027	−0.072	0.032	0.023	−0.059	0.081	0.071	−0.048	0.117*	0.086	−0.029
	14	−0.013	0.065	0.090	0.022	0.097*	−0.031	0.054	−0.059	−0.102*	0.059	0.003	0.065
	15	0.012	0.061	0.046	−0.004	0.007	−0.063	0.003	−0.081	−0.036	0.062	0.034	0.045
	16	0.003	0.055	0.044	0.004	0.036	0.006	0.071	0.059	−0.002	0.092	0.037	−0.017
	17	−0.081	0.061	0.016	0.041	0.077	−0.047	0.041	0.013	−0.010	0.032	0.011	−0.006
	18	−0.004	0.076	0.014	0.050	0.015	−0.034	−0.001	0.053	0.033	0.116*	−0.017	−0.016
Natural sound	19	0.057	0.068	0.065	−0.117*	0.061	−0.052	0.055	−0.088	−0.009	−0.077	0.114*	−0.059
	20	0.069	0.109*	0.071	−0.012	−0.059	−0.035	0.048	−0.053	0.058	0.031	0.104*	0.010
	21	0.117*	0.080	0.097*	−0.076	0.083	0.035	0.066	−0.090	0.015	−0.086	0.044	0.022
	22	0.087	0.138**	0.068	0.063	0.057	0.061	0.012	−0.073	0.021	−0.022	0.048	0.009
	23	0.108*	0.051	0.060	0.002	0.108*	−0.002	0.047	−0.137**	−0.046	−0.023	0.104*	0.011
Livestock sound	24	0.062	0.000	0.074	0.022	−0.083	0.000	0.083	0.105*	−0.003	0.056	0.060	0.005
	25	0.070	−0.050	0.027	−0.005	−0.044	−0.024	0.089	0.082	0.003	0.016	0.046	0.028
	26	0.049	−0.050	0.043	−0.017	−0.055	−0.011	0.062	0.032	0.005	0.062	−0.022	0.056
	27	0.079	0.062	−0.008	0.001	0.050	−0.049	0.042	−0.071	−0.044	−0.067	0.004	0.055
	28	0.075	0.016	−0.010	0.009	−0.025	−0.045	0.111*	0.072	0.038	−0.003	0.058	0.007
Melody	29	0.075	0.017	0.069	0.047	0.024	0.058	0.019	−0.038	−0.012	−0.060	0.032	0.048
	30	0.008	0.058	0.080	−0.021	0.050	0.032	0.001	−0.039	−0.025	−0.009	−0.006	0.136**
	31	0.099*	0.003	0.085	0.010	0.042	0.004	0.002	0.032	−0.027	0.042	0.003	0.051
	32	0.037	−0.021	0.061	−0.061	0.047	−0.031	0.010	0.011	−0.065	−0.001	−0.019	−0.008
	33	−0.037	0.035	−0.039	−0.014	0.053	−0.075	0.039	−0.002	−0.055	0.061	−0.032	−0.071
	34	0.053	0.007	0.071	0.021	0.103*	−0.015	−0.003	0.030	−0.077	0.066	−0.012	0.008
	35	0.015	0.005	0.084	−0.049	0.072	−0.059	0.026	0.068	−0.137**	0.075	0.027	−0.102*

Spearman correlation coefficient significance (* for P ≤ 0.05, **for P ≤ 0.01).

**TABLE 3:** Correlation between public landscape space preference and soundscape preference 2 (code is shown in Table 1).

Sound classification		22. Purpose of going to urban park					27. Preferred public landscape under pandemic situation									
		green space under pandemic situation														
		View	Rest	Play	Recuperate	Motion	Business zone	Street	Square	Stadium and	Lawn	Woods	Forest	Rivers	Sea	Field
Traffic sound	1	−0.136**	0.007	−0.003	−0.038	0.158**	−0.094	−0.058	−0.087	−0.033	0.028	0.178**	0.081	0.101*	0.013	0.110*
	2	−0.125**	0.001	−0.004	−0.035	0.139**	0.031	0.064	−0.003	−0.001	−0.017	0.059	−0.044	−0.053	−0.088	−0.060
	3	−0.043	0.048	0.061	0.011	0.022	0.034	0.024	−0.036	−0.001	0.018	0.030	−0.073	−0.069	−0.087	−0.064
	4	−0.063	−0.008	0.091	−0.032	0.105*	−0.061	−0.007	−0.051	0.012	0.037	0.089	0.007	0.067	−0.028	0.106*
	5	−0.030	0.058	0.057	0.013	−0.010	0.092	0.074	−0.017	−0.012	0.050	0.081	0.029	−0.040	−0.083	−0.052
	6	0.008	0.058	0.066	0.020	−0.039	0.106*	0.082	0.028	0.000	0.020	0.031	0.001	−0.028	−0.043	−0.051
Mechanical sound	7	−0.041	0.017	−0.005	0.073	0.037	−0.034	0.013	−0.034	−0.009	0.055	0.097*	−0.073	−0.079	−0.117*	−0.040
	8	−0.052	−0.003	0.070	0.094	0.046	0.000	0.012	−0.032	0.008	0.122*	0.116*	0.016	0.081	0.040	0.024
Human activity sound	9	−0.030	−0.054	0.139**	0.026	0.081	−0.068	−0.030	−0.040	−0.011	0.073	0.120*	0.083	0.054	−0.030	0.073
	10	−0.027	−0.062	0.097*	0.021	0.048	−0.032	0.029	−0.011	−0.039	0.075	0.116*	0.024	0.068	−0.039	0.087
	11	0.030	−0.029	0.028	0.078	0.040	0.000	0.064	0.028	0.064	0.070	0.110*	0.043	0.037	0.007	0.049
	12	−0.027	−0.040	0.070	−0.022	0.139**	−0.207**	−0.094	−0.074	−0.050	0.062	0.137**	0.064	0.076	−0.021	0.139**
	13	−0.024	−0.023	0.044	0.010	0.039	−0.135**	−0.072	−0.045	−0.041	0.081	0.094	−0.011	0.019	−0.060	0.030
	14	0.057	0.013	0.051	0.015	−0.024	0.047	0.004	−0.006	0.020	0.012	0.002	−0.073	−0.016	−0.008	−0.070
	15	0.057	−0.057	0.065	−0.009	0.028	0.004	−0.058	−0.025	−0.025	−0.023	0.038	0.019	0.012	−0.035	−0.002
	16	0.071	−0.042	0.002	0.051	0.024	0.022	0.020	0.022	0.046	0.018	0.047	−0.016	−0.009	−0.077	−0.019
	17	0.071	−0.042	0.060	0.016	−0.044	0.001	0.003	0.003	0.006	0.024	0.031	−0.055	0.034	−0.047	0.001
	18	0.049	−0.027	0.006	0.028	0.018	0.016	0.013	0.012	0.052	0.031	0.020	−0.089	−0.005	−0.051	−0.033
Natural sound	19	−0.013	−0.054	0.137**	0.010	0.060	−0.098*	−0.135**	−0.113*	−0.038	0.088	0.087	0.203**	0.132**	0.063	0.114*
	20	−0.037	−0.023	0.077	0.031	0.059	−0.062	−0.098*	−0.019	−0.042	0.098*	0.136**	0.163**	0.094	0.053	0.032
	21	0.014	−0.078	0.135**	0.065	0.068	−0.077	−0.093	−0.082	−0.053	0.125**	0.124**	0.217**	0.179**	0.100*	0.141**
	22	−0.035	−0.045	0.063	0.095*	0.078	−0.057	−0.099*	−0.042	−0.028	0.130**	0.104*	0.082	0.144**	0.048	0.039
	23	−0.022	−0.061	0.110*	0.023	0.127**	−0.102*	−0.106*	−0.101*	−0.072	0.114*	0.115*	0.177**	0.143**	0.069	0.146**
Livestock sound	24	−0.108*	−0.049	0.071	0.095*	0.195**	−0.107*	−0.036	−0.015	−0.049	0.137**	0.145**	0.150**	0.127**	−0.045	0.117*
	25	−0.046	0.011	0.084	0.102*	0.195**	−0.078	−0.069	−0.043	−0.043	0.105*	0.131**	0.170**	0.121*	−0.005	0.130**
	26	−0.080	0.007	0.055	0.140**	0.186**	−0.097*	−0.036	−0.051	0.011	0.069	0.143**	0.174**	0.126**	0.036	0.126**
	27	−0.028	−0.082	0.128**	0.052	0.148**	−0.170**	−0.079	−0.036	0.019	0.140**	0.218**	0.141**	0.146**	0.011	0.132**
	28	−0.071	−0.080	0.098*	−0.003	0.187**	−0.166**	−0.069	−0.041	−0.065	0.188**	0.220**	0.204**	0.167**	0.007	0.125**
Melody	29	−0.043	−0.067	0.171**	0.075	0.048	−0.027	−0.026	−0.058	−0.060	0.132**	0.167**	0.134**	0.181**	0.093	0.071
	30	−0.020	0.113*	0.097*	0.064	0.023	0.093	0.042	−0.039	0.003	−0.060	−0.051	−0.006	0.019	0.040	0.005
	31	−0.022	0.002	0.148**	0.073	−0.018	−0.008	0.020	−0.011	−0.030	0.007	0.018	0.022	0.069	−0.042	0.050
	32	−0.053	0.033	0.115*	0.019	0.027	−0.020	0.037	−0.078	−0.021	0.061	0.027	0.009	0.040	−0.071	0.031
	33	0.033	−0.018	0.039	−0.005	0.014	0.015	−0.002	−0.007	−0.015	0.001	0.005	−0.103*	−0.046	−0.110*	−0.090
	34	−0.038	0.044	0.178**	0.047	0.021	0.006	0.038	−0.037	−0.006	0.053	0.028	0.061	0.122*	−0.025	0.069
	35	0.064	−0.028	0.106*	−0.034	−0.007	−0.111*	0.097*	0.104*	−0.033	0.061	0.066	0.020	0.026	−0.140**	0.050

Spearman correlation coefficient significance (* for P ≤ 0.05, * * for P ≤ 0.01).

In the selection of landscape structural design, those who use walkways have a relatively low preference for the sound of wind blowing leaves, while respondents who prefer water pavilions, waterside trails, and forest paths have a higher preference for the sound of wind blowing leaves. Individuals who prefer forest paths have a relatively high preference for the howling of the wind, while those who favor sports and benches have a low preference. In terms of water landscape preferences, those who favor the sea have a higher preference for pop music while individuals who prefer fountains are more receptive to the sound of square dancing.

Data analysis shows that wearing a mask has a significant impact on the correlation of soundscape preferences. The results show that if low numbers of people wear masks while viewing the scenery, there is a lower preference for the sounds of traffic, mechanical sounds, and stranger calls, babies crying, coughing, and sneezing. Conversely, people who wear mask when viewing the scenery will give a relatively lower soundscape evaluation on the noise.

On the whole, soundscape preference is inseparable from the environmental conditions and the atmosphere created by the public landscape space. Visual landscape preference indirectly affects the overall soundscape preference through the perceived incidence and loudness of sound (Liu et al., 2019b). The results verify that people’s subjective evaluations of soundscapes are closely related to the soundscape conditions that typically appear in specific environments and the usual atmosphere. In the case of a long-term pandemic, public landscape designers need to consider the impact of wearing masks on people’s perception of soundscapes.

### Influence of Living Conditions on Soundscape Preference in the Post-pandemic Era

The results (Table 4) show that the greening rate (19), the distance (20) of the green space, and the frequency of going to the public green space are related to the purpose (21) and the degree of soundscape preference. Based on the correlation results obtained, it can be inferred: in terms of greening rate, the higher the surrounding greenery, the higher the degree of people’s preference for industrial noise, farming work, chicken crows, and vehicle engine noise. In terms of green space distance, the closer the residents are to the green space, the higher the tolerance for strangers’ calling, people clearing their throats, and ambulances. In terms of the frequency of going to the green space, the higher the frequency of going to the green space, the more people like the sound of livestock. This may mean that green spaces can bring people closer to nature, therefore people prefer animals more. In general, the results of this study combined with the theory of stress reduction and the theory of attention recovery (Wen, 1989), can further speculate that the surrounding greening rate can relieve people’s stress and fear in the post-pandemic era in the city.

**TABLE 4:** Correlation between personal living conditions and soundscape preference (code is shown in Table 1).

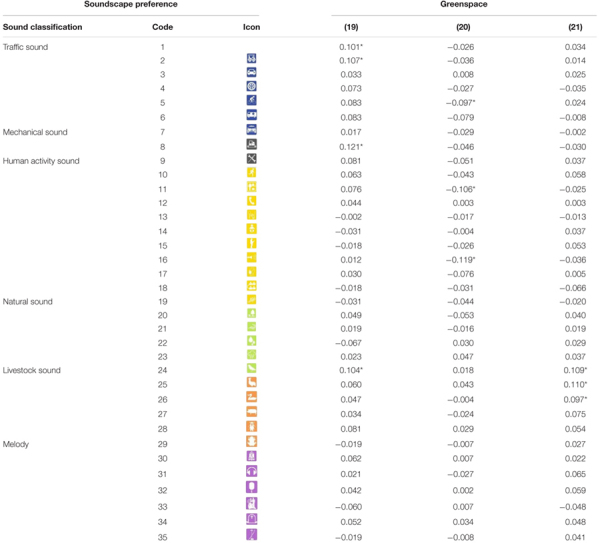

Spearman correlation coefficient significance (* for P ≤ 0.05).

### The Influence of Other Factors on the Soundscape in the Post-pandemic Era

#### Personal Characteristics

##### Gender

**FIGURE 5:**
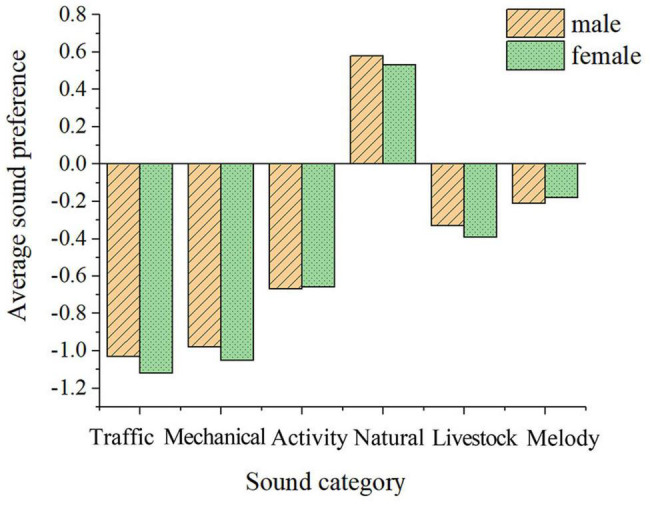
Average soundscape preference of people of different genders.

This study found that gender has a negligible effect on soundscape preference in the post-pandemic era, which is consistent with the results of previous studies (Yu and Kang, 2010). The results (as shown in Figure 5) show that men have a lower preference for square dance sounds than women, but they have a higher acceptance of howling winds. In China, square dance activities are dominated by women, and women are more sensitive and emotional than men, this also provides an explanation to why woman’s soundscape preference for the wind and whistling sounds is lower for men (Meng et al., 2010).

##### Age

**FIGURE 6:**
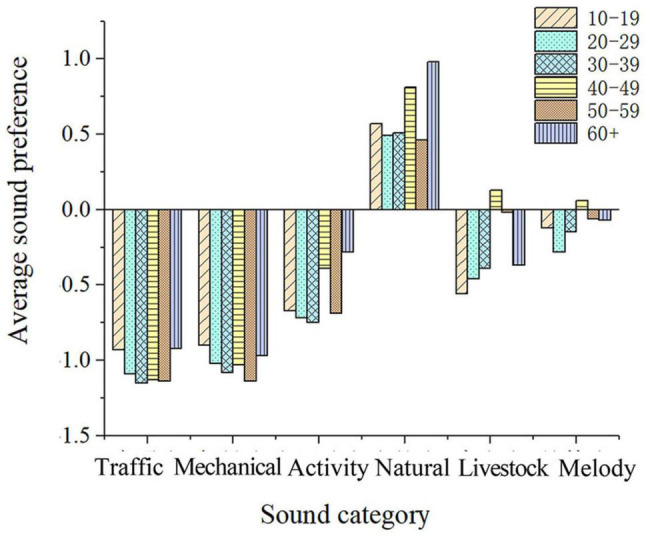
Average soundscape preference of varying age groups.

According to related studies, age and soundscape preference are significantly correlated (Meng et al., 2010; Aletta et al., 2019). It can be seen from Figure 6, that the respondents’ age is positively correlated with natural sounds such as the sound of people’s activities in the post-pandemic era, the sound of wind and leaves, running water, domestic animals, festival songs and dances, and the sound of local folk music; but it is negatively correlated with pop music. This suggests that as residents grow older, their evaluation of the soundscape of human activities, natural sounds, domestic animal sounds, and music increases while their auditory preferences of pop music decreases. These results are consistent with the results of previous studies (Zhou et al., 2012). This result suggests that the older adults favor children and conversation over other auditory stimuli in the post-pandemic era.

##### Occupation

**FIGURE 7:**
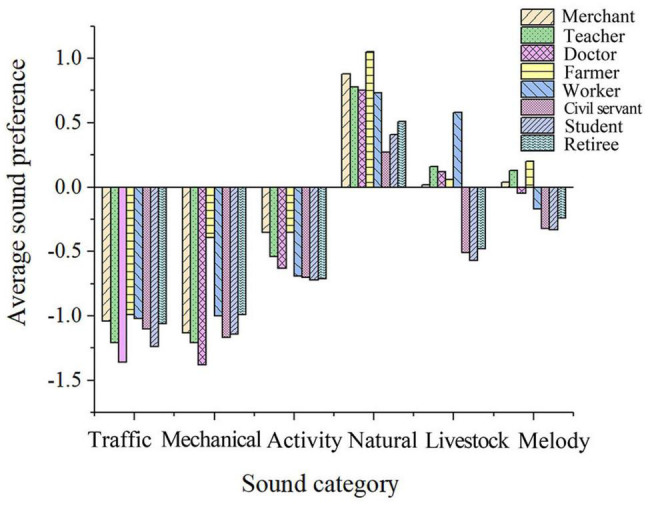
Average soundscape preference of different occupational groups.

According to the correlation analysis, it is found that occupational factors have a significant impact on the subjective evaluation of soundscape preferences. As shown in Figure 7, doctors, retirees, and teachers have relatively high preferences for human activity sounds, natural sounds, and music, while workers and government workers have low preferences for human activity sounds and natural sounds. Retirees have the highest preference for the sound of wind blowing leaves. Students have a lower preference for the sound of domestic animals, babies crying, local folk music, and square dancing. The results suggest that professionals who have more contact with people—such as doctors and teachers—generally have a higher tolerance for acoustic disruptions than other occupations and that migrant workers, government workers, and students have fewer soundscape preferences in the post-pandemic era.

##### Faith

**FIGURE 8:**
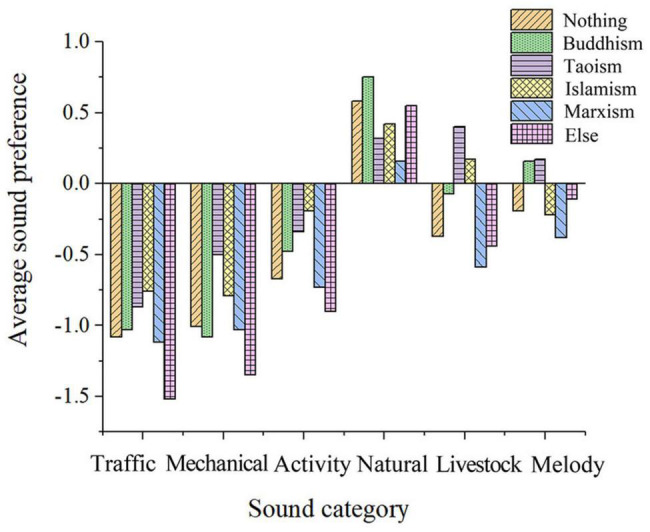
Average soundscape preference of people of different beliefs.

As shown in Figure 8, Islamic people have a lower degree of preference for the sound of pigs, cattle, and sheep, while Marxists have a lower degree of preference for the sound of howling wind. Respondents who do not identify as religious have a highest preference for popular music while those who identify as Buddhists and Marxists have the lowest. Islamic practitioners’ preference for funeral music is higher than that of other religious believers. Religious ideology impacts the degree of soundscape preference; it is possible that the living habits and specific religious belief of an individual can indirectly affect the evaluation of soundscape in the post-pandemic era.

##### Education

**FIGURE 9:**
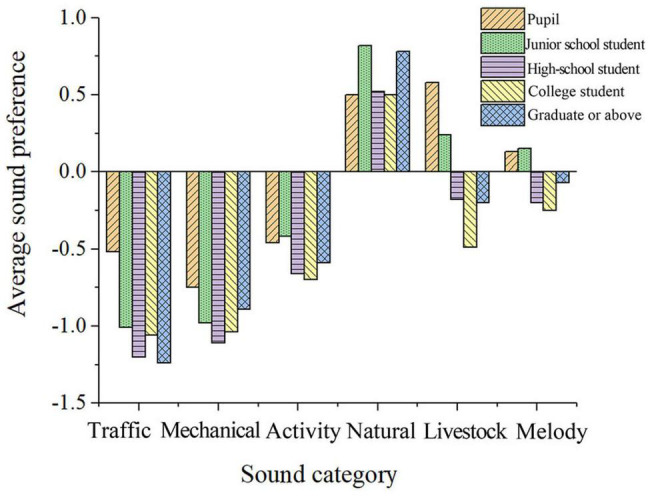
Average soundscape preference of differently educated people.

As shown in Figure 9, the level of education is negatively correlated with the soundscape preferences of square dances, livestock sounds, wedding music, children’s frolicking, babies crying, farming operations, and vehicle engine sounds in the post-pandemic era. This study supports the conclusion that the higher the level of education, the lower their soundscape preferences and comfort (Meng et al., 2010).

##### Marriage

**FIGURE 10:**
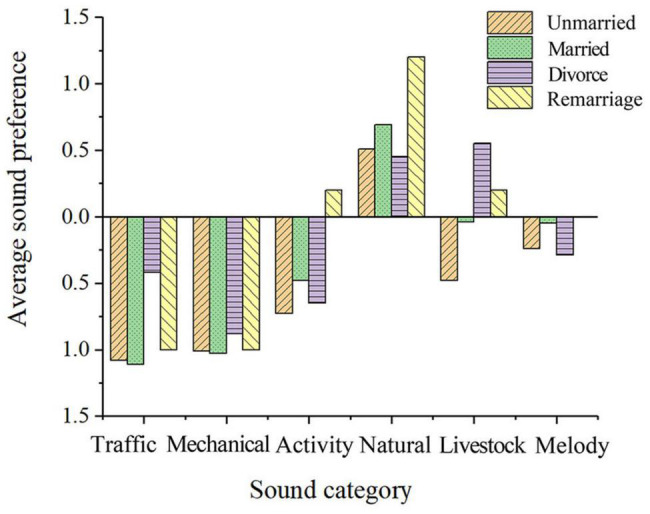
Mean value of soundscape preference of different marriage groups.

As shown in Figure 10, married people have a higher preference for conversation, children’s frolicking, and baby crying, while unmarried people have a lower preference for cock crowing in the post-pandemic era. The surveyed results suggest married people are more tolerant of crowds and children and that they are more inclined to social gatherings than unmarried people.

##### Number of Children

**FIGURE 11:**
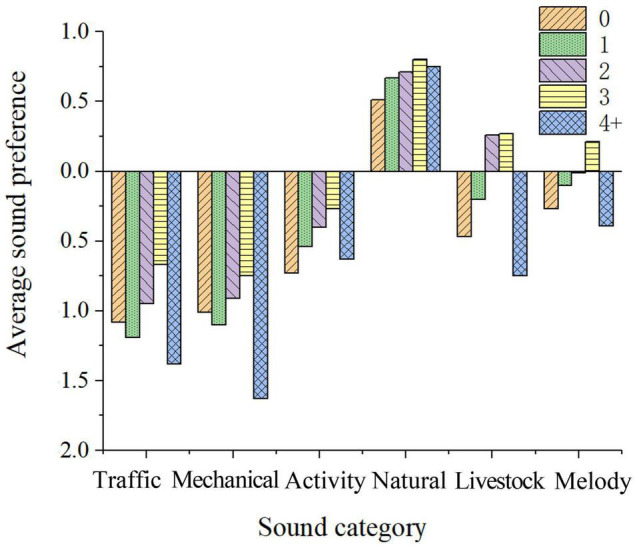
Sound preference of families with different numbers of children.

As shown in Figure 11, with the increase in the number of children, people’s preference for children’s frolicking, human speech, domestic animal sounds, square dances, and local folk music increases. This result may reveal that families with more children are more likely to participate in social activities in the post-pandemic era.

##### Family Housing Situation

**FIGURE 12:**
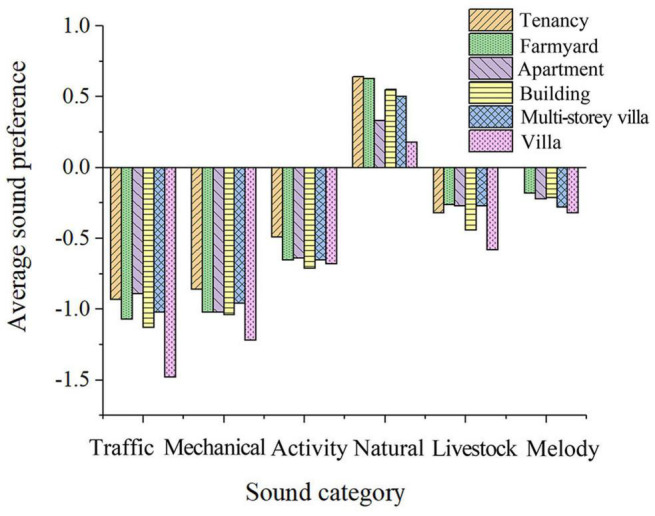
Sound preference of people with varying conditions of housing.

According to the correlation analysis, the annual household income has no significant impact on the soundscape preference evaluation. Still, the family housing situation is correlated with soundscape preferences (Figure 12). The results show that the better housing conditions are, the lower the tolerance for acoustic disturbances. However, individuals who rent houses and villas have a high degree of preference for children’s frolicking, in contrast, residents living in white-collar apartments and high-rise buildings have less preference for children’s frolicking. It may be revealing that young working-class people have increasing economic and reproductive pressures, and have a lower preference for children in the post-pandemic era.

Overall, under the impact of COVID-19, in terms of personal characteristics, family environment, age, occupation, education, and family housing are the main factors affecting soundscape preference. Gender, beliefs, marriage, and other factors have a certain degree of influence on auditory preference. Physical condition, BMI, annual family income, and the number of people in the family have no significant impact on soundscape preferences in the post-pandemic era.

#### Life State Under the Impact of the Pandemic

##### The Impact of Vaccination

To explore whether the COVID-19 vaccination impacts the preference of the soundscape, this manuscript analyzes the correlation coefficient and significance level of the Chinese respondents’ vaccinations of the vaccine (12) and the preference of the soundscape (Table 5).

**TABLE 5:** Correlation between life state and soundscape preference in the post− (code is shown in Table 1).

M	Life state					
Code	(10)	(11)	(12)	(16)	(17)	(18)
1	−0.028	−0.083	−0.043	0.087	−0.077	0.049
2	0.003	0.006	−0.071	−0.009	−0.115*	−0.012
3	−0.004	−0.083	−0.102*	−0.024	−0.068	−0.043
4	−0.064	−0.051	−0.082	0.036	−0.095*	0.006
5	0.033	−0.097*	−0.056	0.039	−0.019	0.015
6	0.037	−0.078	−0.075	0.017	−0.011	0.032
7	−0.021	−0.162**	−0.086	−0.001	−0.060	−0.025
8	−0.077	−0.128**	−0.150**	0.094	−0.018	0.013
9	0.025	−0.007	−0.133**	0.021	−0.091	0.139**
10	−0.003	−0.032	−0.147**	−0.052	−0.195**	0.135**
11	−0.015	−0.043	−0.088	0.008	−0.082	0.071
12	0.018	−0.043	−0.068	−0.004	−0.150**	0.083
13	0.018	−0.052	−0.084	0.027	−0.085	0.021
14	−0.024	−0.097*	−0.098*	0.032	−0.050	0.081
15	−0.040	−0.043	−0.075	0.019	−0.116*	0.064
16	−0.045	−0.085	−0.061	0.036	−0.060	0.039
17	0.010	−0.057	−0.114*	−0.014	−0.065	0.024
18	−0.040	−0.079	−0.073	0.046	−0.033	0.005
19	0.042	0.043	0.005	0.013	−0.028	0.026
20	−0.002	0.031	−0.047	0.042	−0.052	0.086
21	0.005	0.002	−0.090	0.092	−0.063	0.027
22	−0.005	−0.047	−0.073	0.105*	0.022	0.032
23	0.052	0.013	−0.059	0.013	−0.071	0.017
24	−0.002	−0.016	−0.057	0.067	−0.074	0.039
25	−0.019	−0.028	−0.087	0.052	−0.075	0.049
26	−0.075	0.006	−0.065	0.108*	−0.040	0.047
27	0.010	−0.021	−0.039	0.026	−0.058	0.042
28	0.044	0.017	−0.095	0.056	−0.053	0.078
29	−0.061	−0.076	−0.035	0.106*	−0.022	0.025
30	−0.012	−0.018	0.000	−0.020	−0.065	0.093
31	0.055	−0.069	−0.071	0.063	−0.114*	0.104*
32	0.054	−0.026	−0.035	0.018	−0.137**	0.065
33	0.014	−0.059	−0.011	0.042	−0.019	−0.031
34	0.022	−0.028	−0.057	0.067	−0.087	0.088
35	0.107*	−0.007	−0.044	−0.023	−0.103*	0.032

Spearman correlation coefficient significance (* for P ≤ 0.05, * * for P ≤ 0.01).

*M, sound type.*

(10) Compared with before the pandemic, has your BMI changed?
(11) Have you been infected with COVID-19?
(12) Have you and your family or friends infected with COVID-19?
(17) Compared with before the pandemic, do the soundscapes make you feel more nostalgic?

The study found that people have a lower preference for ambulances and construction noise regardless of whether they are vaccinated with the vaccine. The pandemic has caused many deaths and illness and has caused an increase in people’s sensitivity to grief events and noise. Interestingly, the injection of the vaccine brings people a sense of psychological security. The results show that vaccinated people have a slightly higher preference for road noise, handicraft noise, throat coughing, live street performances, and wind whistling sound than unvaccinated individuals. Furthermore, they have a slightly higher preference for human footsteps as well as for having conversations in the post-pandemic era.

##### Influence of Hometown Attachment and Happiness

After investigating whether respondents reside in their hometown, their attachment to their hometown, and the happiness in their life, this article finds that soundscape preference is somewhat affected by homesickness and joy. The results show that people who reside in their hometown have a higher preference for the sound of heavy rain, livestock, and temple bells, while respondents who feel nostalgic toward their hometown have a higher preference for the sound of vehicle engines, human conversations, children’s frolicking, festive singing, and dancing. Individuals who identify as having high happiness in their lives have a high degree of preference for wedding music and square dance sounds. Their preference for the sound of human footsteps, human conversation, festival singing, dancing, and local music and art is also higher than those with lower happiness levels. This is consistent with previous studies which have suggested that people with higher happiness are more likely to report positive soundscapes. Conversely, people with lower happiness levels are more likely to have negative attitudes toward the sound environment (Aletta et al., 2019). It can be inferred that happiness and hometown attachment can increase an individual’s soundscape evaluation in the post-pandemic era.

##### The Influence of Socioeconomic Status on the Perception of Soundscape

The socioeconomic status of an individual or family is often considered comprehensively based on factors such as income, education, and occupation (Wen, 1989). Evidence suggests that the better the family’s housing situation, the lower the degree of people’s preference for noise such as traffic sounds. In the post-pandemic era, it is worth noting that among those with a high socioeconomic status, individuals who live in villas and those with extreme affluence have a high degree of preference for the sound of children’s frolicking. In contrast, residents who live in white-collar apartments and high-rise buildings have less preference for the noises of children’s frolicking. The higher the level of education, the higher preference for most music and human activity sounds—especially children’s frolicking and babies crying—while preference for livestock sounds and traffic noise is lower. In terms of occupation, doctors and teachers have a relatively high preference for human activity sounds, natural sounds, and music. Government workers have a low preference for human activity sounds and natural sounds.

Overall, It can be inferred that the higher the socioeconomic status, the lower the tolerance for noise. With the different attributes of different occupations, the soundscape preferences of people with high socioeconomic status are more targeted, which is related to the nature of their careers in the post-pandemic era.

#### Soundscape Preference of Vulnerable Groups

##### Soundscape Preference of the Elderly

The elderly people are prominent participants in the public landscape space, and their preference for soundscape landscape is a critical indicator of soundscape optimization. At present, scholars have researched the auditory needs of the elderly and found that they prefer natural sounds and stimulating music (Wang and Kang, 2020). However, their preference is often affected by the type of activity in which they are participating. For example, when playing ball or sitting they tend to prefer sound while when they are playing or watching chess, they do not want to hear any sounds, including soothing music. On the whole, amongst the elderly there is a significant positive correlation between the general sound environment and the preferences for additional natural sounds (Wang et al., 2020). This study shows that people aged 50–59 and over 60 have a high preference for human activity sounds, natural sounds, domestic animal sounds, and music. They have a low preference for pop music sounds under the influence of the pandemic.

##### Soundscape Preferences of Teenagers and Children

Studies have shown that the soundscape of urban parks can promote children’s psychological and physiological recovery (Shu and Ma, 2020). Therefore, the soundscape preference of teenagers and children is essential when considering soundscape design in environments with many adolescents. The results of this study show that teenagers have a higher preference for the sound of wind blowing leaves, running water, bird songs, festive singing and dancing, and pop music. Under the influence of the pandemic, they have a high preference for the sounds of footsteps, conversations, children’s frolicking, and babies crying, besides, the soundscape preference for the sounds of chickens, domestic animals, cicadas, frogs, and square dancing are relatively low. This suggests that adolescent soundscape preferences are biased toward soothing natural sounds and modern music and are not interested in human activities, domestic animal sounds, and natural sounds and traditional music in the post-pandemic era.

##### Soundscape Preferences of Unhealthy and Obese People

The survey results show that, under the influence of the pandemic, people in poor physical condition (9) have a higher preference for the sound of wind blowing leaves, while obese people (10) have a higher preference for square dancing. This suggests that unhealthy people desire the related healing effect of natural sound, while obese people have the desire to do outdoor-related exercise under the influence of the pandemic.

In general, the elderly people, adolescents, and unhealthy people have a higher preference for natural sound, especially the elderly. The soundscape preferences of teenagers and children are more inclined toward popular music. Obese people have a higher preference for square dancing—perhaps due to their desire for exercise. The Soundscape preferences of disadvantaged groups are affected by their own psychological and physical requirements, and the soundscape can alleviate their physical and mental needs to a certain extent in the post-pandemic era.

### Automatic Linear Model of Soundscape Preference in the Post-pandemic Era

**FIGURE 13:**
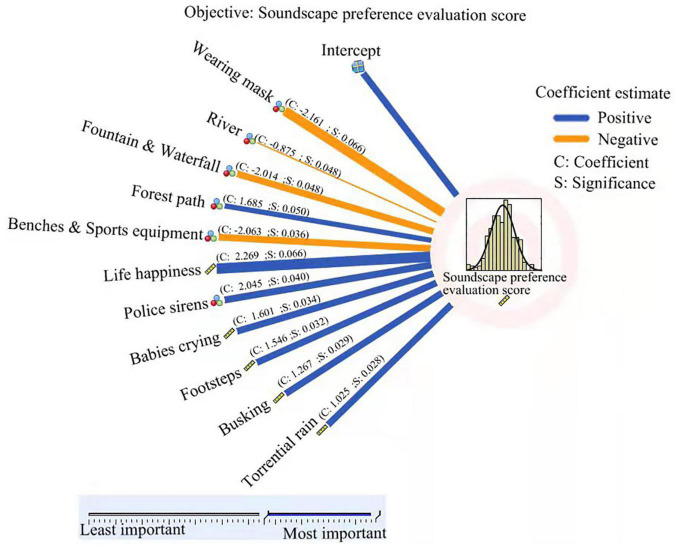
Automatic linear model of soundscape preference evaluation score as target.

To further analyze the preference of soundscape, the automatic linear model is applied with SPSS26.0, andsoundscape preference evaluation is divided into the target variable. The relationship between 52 other predictive variables is shown in Figure 13.

In the overall factors, happiness and the soundscape preference for the personal assessment show a strong positive correlation. It can be assumed that if the pandemic reduces people’s happiness, it will directly force the soundscape preference to decrease, which indicates that the pandemic has a weakening effect on soundscape preference. In addition, it is found that people who like the landscape of the forest path, more sensitive to the sound of police sirens, babies crying, footsteps, busking, and torrential rain have a higher soundscape preference evaluation score in the post-pandemic era.

In particular, it can be inferred that people who are unwilling to wear a mask to enjoy scenery have a relatively higher total subjective evaluation score. This suggests that wearing masks directly reduces people’s evaluation of soundscape, revealing that the epidemic affects soundscape evaluation in another way. In addition, it is found that people who like water environment—with preference for rivers, fountains waterfalls—had a negatively correlated soundscape evaluation in the post-pandemic era. This may be because people who like water scenes tend to be quiet and reflective, rather than disturbed by other sounds. Moreover, this study found that people who like do leisure sports—with preference for benches and sports equipment—had a negatively correlated soundscape preference evaluation. This may be because people who enjoy leisure and exercise do not like to be disturbed, so they have lower soundscape evaluation score.

## Conclusion

This study used the subjective evaluation method to conduct an online questionnaire survey in 29 Chinese provinces with high infection rates. By analyzing the correlation and reasons for different landscape and soundscape preferences, it was found that people with different attributes have different perceptions of landscape and soundscape in the post-pandemic era. The main findings are summarized as follows:

### Direct Impact of Pandemic Prevention Measures on Soundscape Evaluation

#### Wearing Masks

Among common quarantine measures during the pandemic, wearing masks significantly reduced soundscape perception ratings, especially for sounds that people did not like before the pandemic, such as traffic, babies crying, construction, and stranger calls. People also show great aversion to audible hints of possible infection, such as clearing their throats and sneezing.

#### Vaccine

People who have been vaccinated are more tolerant of various noises. Their preference for road noise, handicraft noise, people clearing their throats and coughing, live street performances, and gale screaming is slightly higher than that of unvaccinated individuals. Their preference for human footsteps and human conversations is higher, revealing that they showed relative trust and security toward strangers.

### Main Influencing Factors of Soundscape Preference in the Post-pandemic Era

#### Main Influencing Factors

The study confirms that the overall soundscape preferences of urban residents in public spaces were affected by the pandemic. The results revealed natural sound and music have the highest overall impact on soundscape preference, whilst the overall value of soundscape preference for traffic sounds is the lowest. Among the natural sounds, the top 3 preference are natural running water, wind blowing leaves, and bird song.

#### Impact of the Epidemic

Under the pandemic situation, people show high sensitivity to emergencies and deaths, which affects the evaluation of soundscape preferences to a certain extent. Ambulance sounds, funeral music, and police car sirens accounted for higher aversion than road traffic noise which are usually the least preferred.

#### Impact of the Landscape

Under the influence of the pandemic, people’s subjective evaluation of soundscape is closely related to the landscape. Public landscape space type, landscape structure design, and water landscape are the three most important landscape factors that affect the soundscape preference, which reaffirms that people can indirectly affect the overall sound landscape preference through the spectrum and loudness of perceived soundscape.

#### Impact of the Greening Rate

In the post-pandemic era, the high correlation of data analysis shows that increasing the greening rate can improve the evaluation of sound landscape to a certain extent, and the closer the distance between residents and green space, the higher tolerance of noise to a certain extent. Moreover, people who visit green Spaces more often are likely to enjoy animals more.

### Impact of Individual Factors on Soundscape Evaluation Under the Pandemic Influence

#### Main Influencing Factors

Among personal characteristics, age, occupation, education, and family housing are the main factors that affect soundscape preference. In contrast, gender, belief, marriage, and other factors have a secondary degree of influence on auditory preference. Physical condition, BMI value, annual family income, and the number of older people in the family do not affect soundscape preferences.

#### Disadvantaged Groups

The soundscape preferences of disadvantaged groups are often affected by their own psychological and physical needs. Some elderly people are fonder of physical activities, natural sounds, livestock sounds, and music. They express their yearning for the natural environment and lively crowds. Children’s soundscape preferences tend to be soothing natural sounds and trendy music which is more energetic. Obese people have a higher preference for square dancing due to their own fat loss needs in the post-pandemic era.

#### Life Happiness

In the overall subjective factors, life happiness and the soundscape evaluation for the personal assessment show a strong positive correlation.

## Discussion

Based on the above conclusions, in this section, we presented some discussions on applying the findings to the design recommendations of urban public space in the post-pandemic era.

### Design Recommendations of Landscape to Enhance Soundscape Experience in the Post-pandemic Era

#### Overall Landscape Design

The study found that the subjective evaluation of soundscape is closely related to the acoustic conditions of the place and the landscape. Within the overall landscape design, the integration of soundscape must be fully considered; a harmony can be created through architectural landscape structural design, waterscape, plant design, as well as other methods. The soundscape interacts with different landscape to form a good environment of audio-visual integration. Secondly, it is necessary to enrich the space with activities to guide people’s behavior and reduce people’s perception of unfavorable soundscape factors.

#### Green Environment Design

Studies have found that the greening rate, the distance to green space, as well as the purpose and frequency of public green space use, impacts soundscape preference. Previous studies have also found that increasing exposure to green plants can reduce stress, thereby affecting the soundscape evaluation. Green landscape, walkways, and trails can be set up to produce the sound of wind blowing leaves, thereby triggering people’s resonance with natural sounds. However, it is not suitable to set up a natural soundscape beside promenade, sports equipment, benches, and other sports and leisure facilities, which mainly emphasis the soundscape of human activities.

#### Blue Environment Design

According to the research results, people show a greater willingness to approach blue environment in the post-pandemic era. Landscape design should increase the capacity of blue environment, as far as possible to make people close to or watch the water, fountains or listen to the sound of rain. If some devices can be properly set up, the water can be enlarged or activated, which will help people relax, more contact with nature, and improve the scenery experience.

#### Tour Space Design

This study found that the comfort of wearing a mask has a significant impact on evaluating soundscape preference. People who have a worse viewing experience when wearing a mask have a lower preference for traffic sounds, mechanical sounds, and human activities. On the contrary, people who wear masks are more inclined to traffic sounds, mechanical sounds, and human activity sounds. Wearing masks in the post-pandemic era has become the norm. Considering the characteristics of individuals with negative experiences of wearing masks, the distance between people should be considered in the design of tour routes and transition space should be constructed to form the appropriate distance between individuals, thereby reducing the epidemic impact on human activities.

### Soundscape Construction of Caring for Vulnerable Groups

#### Soundscape Design for Teenagers and Children

According to the data analysis of the survey, families with many children are averse to police sirens, popular music, and funerals; they prefer the sound of human activities, nature, domestic animals, and festive sounds. This shows the importance of sound landscape design for children’s activity space design. To make children have a better experience, designers should create a natural and beautiful soundscape environment while fully integrating natural elements into the site design. Natural materials can be used to make game facilities and environments that are esthetically pleasing. Soundscape design is not limited to traditional forms and can include direct sounds, such as horns, background music, or sounds produced by the natural environment, such as water, wind, insects, and birds. Unique and exciting shapes and sounds can create a compelling space for children to play.

#### Soundscape Design for the Elderly

According to questionnaire analysis, it is understood that families with many older people do not like to rest; instead, they want to exercise. They also like to go to places with mountains, rivers, and forests. Still, designers should not only create landscape that the older people prefer but must also take their physical condition and safety into consideration. In this regard, we can put forward the following landscape suggestions:

① Construct barrier-free design and transform the road system. For example, set up safe passages for the elderly people on the main routes, increase safety handrails, non-slip pavement, and warning signs.

② Rest areas design for the elderly people. Even though the elderly people may not like to rest very much, they are prone to fatigue when walking outside. Therefore, constructing seats in activity spaces is necessary to allow them to perform rest and setting up trash cans near the seat to facilitate the collection of waste. Developing corresponding service facilities will allow comfort and security to be brought to the elderly people.

③ Regarding the layout of outdoor space plants, it is necessary to consider the coordination of the overall layout. Making a reasonable plan for the shape and color of the plants, while considering outdoor attractions that the elderly people enjoy is essential to overall design. The effect of the plants, plant distributions, plant types, and terrace designs can be used to divide the area for the elderly people. Furthermore, different plant areas can be set for the elderly people with different plant preferences and physical health conditions. Waterscapes should also be added near the plant area to play a role in esthetics and adjustment of the microclimate of the area.

#### Soundscape Design for the Disabled and Unhealthy

For people with poor health—including people with physical disabilities—the survey results show that they have no preference for going out and exercising. Still, they favor the sound and melody of square dances. In this case, the scenery for people with poor health is not essential as they do not frequently use the facilities. Instead, a space should be placed a little further way where people can dance. The environment can be equipped with speakers to play music so that it will not be too noisy for those in poor health. For people with hearing disabilities, visual cues such as text, lights and guardrails should be added to ensure their safety and improve their viewing experience as much as possible. These design recommendations could satisfy their soundscape preference and attract them to hang out in the space, which will benefit for their physical and mental health.

### Reflection

Through the research process, the methodology is valid and can be investigated on a variety of people. This article only studies the impact of the COVID-19 pandemic on the soundscape preferences of Chinese urban residents from the perspective of relevance. It does not consider the changes brought by the pandemic comprehensively. Moreover, because this study was based on statistical correlations, conducted in the post-pandemic era after the outbreak was contained, it could not show absolute cause and effect from the pandemic, the correlations shown by certain factors do not rule out the possibility of some kind of coincidence. In the future, the study and discussion can be strengthened to provide more powerful theoretical support for constructing soundscapes in urban environments.

## Limitations and Strengths

This study focuses on social hotspots and the new changes of soundscape preferences of urban residents in China in the post-pandemic era, the limitations and strengths are as follows:

### Limitations

(1) Because soundscape preference is indirectly affected by the pandemic, there are not only two factors (“wearing masks” and “getting vaccinated”) related to pandemic, but in fact, all thoughts and all aspects of life will be affected by the pandemic, indirectly causing unknown influence on soundscape preference. In this context, the purpose and conclusion of this study are uniformly focused on people’s behaviors, habits and preventive measures to find the new soundscape preference, and reveal the links between soundscapes and other aspects in the post-pandemic era.
(2) Because of a lack of related information collection in the pre-pandemic, the research results cannot reveal the changes of soundscape preference during the pandemic process, nor can they fully demonstrate the causal relationship of soundscape preference under the influence of the pandemic. The correlation between the investigated factors can only be demonstrated from the perspective of data analysis.

### Strengths

(1) Compared with other studies on soundscape preference, this study adopted the first plateau period after the outbreak of the pandemic, which can reflect people’s subjective feelings.
(2) In order to maximize the influence of different factors on soundscape preference, this study summarized literature to produce 65 questions to survey the overall soundscape preferences of the respondents on three parts: the basic situation of the respondent (question 1–18), the landscape preference and overall feeling (question 19–30), and the soundscape preferences (question 35–64). Based on the above relatively rich and complete questionnaire structure, the study contributed more convincing conclusions. Although some results inevitably overlapped with relevant studies, it could further verify the conclusions of previous studies.
(3) In terms of research methods, “automatic linear model of soundscape preference evaluation score as target” has been established, the positive and negative correlation intensity of different factors on soundscape preference can be clearly and intuitively observed.
(4) In addition to questionnaire survey and statistical analysis, this study realistically proposed landscape design methods to increase soundscape preference in the post-pandemic era and proposed soundscape design schemes for vulnerable groups.

## Data Availability Statement

The original contributions presented in the study are included in the article/Supplementary Material, further inquiries can be directed to the corresponding author.

## Ethics Statement

Ethical review and approval was not required for the study on human participants in accordance with the local legislation and institutional requirements. The patients/participants provided their written informed consent to participate in this study.

## Author Contributions

JL: develop research ideas, design research scheme, data collection and analysis, main author of the manuscript, and final revision. JX: research scheme, method guidance, and data analysis. ZW: guidance for revision. YC and YG: data sorting and analysis. JR: text modification. All authors contributed to the article and approved the submitted version.

## Conflict of Interest

The authors declare that the research was conducted in the absence of any commercial or financial relationships that could be construed as a potential conflict of interest.

## Publisher’s Note

All claims expressed in this article are solely those of the authors and do not necessarily represent those of their affiliated organizations, or those of the publisher, the editors and the reviewers. Any product that may be evaluated in this article, or claim that may be made by its manufacturer, is not guaranteed or endorsed by the publisher.
