# Supplementary material for: Soundscape Preference of Urban Residents in China in the Post-pandemic Era
Source: Front Psychol. 2021 Dec 22;12:750421. doi: 10.3389/fpsyg.2021.750421 (PMC8727873; doi:10.3389/fpsyg.2021.750421)
Supplement: Supplementary file 1 [file Data_Sheet_1.PDF]

Soundscape Preference of Urban Residents in China Under COVID-19 Situation

Dear madam/sir:

Hello! We are conducting a survey on the public landscape and soundscape preferences of Chinese urban residents under the new crown pandemic.

The survey questionnaire is expected to take you 10 minutes of precious time. We will keep your identity and answers strictly confidential, and the research results will only be used for scientific research.

Thank you very much for your participation!

1. Gender

☐Male 1 ☐Female 0

2. Age 123456

☐10-19 years old ☐20-29 years old ☐30-39 years old ☐40-49 years old ☐50-59 years old ☐60+ years old

3. Occupation 123456

☐Merchant ☐Teacher ☐Doctor ☐Farmer ☐General staff ☐Government staff ☐Student ☐Retiree

4. Faith 012345

☐none ☐Buddhism ☐Taoism ☐Islam ☐Marxism ☐other

5. Education 12345

☐Primary school ☐Junior high school ☐High school ☐University ☐Postgraduate and above

6. Marriage 1234

☐Unmarried ☐Married ☐Divorced ☐Remarry

7. Number of children in the family (-2~2)

☐0 ☐1 ☐2 ☐3 ☐4 or more

8. Number of elderly in family (-2~2)

☐0 ☐1 ☐2 ☐3 ☐4 or more

9. Physical condition

☐Suffering from a major illness ☐Less healthy ☐Normal ☐Healthy ☐Very healthy

10. Compared with before the pandemic, has your BMI changed? (Refer to BIM value "BMI=weight (kg)/height square (meter)")

☐Skinny（<18.5） ☐Normal（18.5-23.9） ☐Overweight（24 到 27.9） ☐Obese（>28）

11. Have you been infected by the coronavirus?

☐I am infected ☐Family member infected ☐Friend infected ☐A colleague or classmate is infected ☐Not infected

12. Have you and your family and friends received the COVID-19 vaccine?

☐I have been injected ☐Family members have been injected ☐Friend has been injected ☐A colleague or classmate has been injected ☐None injected

13. Whether there are flowers, birds, fish, insects and small animals in the home ? 012345

☐None ☐Cat ☐Dog ☐Bird ☐Fish ☐Flowers and plants

14. Family financial status (total annual income) (-2~2)

☐Poor(＜30000） ☐Low-income（30000-80000） ☐Well off（80000-300000） ☐High-income（300000-1000000）

☐Rich（>1000000）

15. Family housing situation

☐ Renting ☐ Farmhouse ☐White-collar apartment ☐High-rise building ☐Multi-storey independent building

☐Villa

16. Is your place residence in your hometown?

☐Yes ☐No

17. Do the soundscapes make you feel nostalgic?

☐Yes ☐No

18. Do the sounds make you happiness in daily life?

☐Very unhappy ☐Less happy ☐General ☐Happier ☐Very happy

19. What is the ecological greening situation around your usual place of residence?（Greening rate）

☐Very bad (<15%) ☐Worse (15%-35%) ☐General (35%-50%) ☐Better (50%-70%) ☐Very good (>70%)

20. The distance to the public landscape green space closest to your address 12345

☐Within 500 meters ☐500 meters-2000 meters ☐2 km-10 km ☐10km-20km ☐More than 20 kilometers

Please choose the “0”:

☐0 ☐1 ☐2 ☐3 ☐4

21. Before and after the pandemic, how often did you go to public green spaces?

☐Increase ☐Constant ☐Reduce

22. Compared to before the pandemic, what was your purpose of going to the city parks and green spaces?

☐Admire the scenery ☐Rest ☐Play ☐Convalescence ☐Fitness ☐Walking pets ☐Family activities

23. Compared to before the pandemic, what is your more ideal public landscape destination?

☐Residential area ☐ City Mall ☐ City scenic area ☐City gym ☐Rural area

24. Compared with before the pandemic, your current life is more inclined (multiple choice)? 12345

☐More depressing ☐More anxious ☐More pressure ☐Calm as usual ☐More pleasant

25. Compared to before the pandemic, what was your mood when going out to enjoy the scenery?

☐Very panic ☐Slight fear ☐Calm and normal ☐ No fear ☐ No fear at all

26. What is your experience of enjoying the scenery while wearing a mask?

☐Very uncomfortable ☐ Less comfortable ☐General ☐More comfortable ☐Very comfortable

27. Compared to before the pandemic, which public landscape do you want to visit more?

☐Business district ☐Street ☐Square ☐Playground ☐Grassland ☐ Forest ☐Mountain forest ☐River ☐Seaside ☐Field

28. Compared with before the pandemic, which landscape sketch do you prefer to stay in?

☐Bench ☐Sport equipment ☐Pavilion ☐gallery ☐Flower stand ☐ Water pavilion ☐Waterside trail ☐Forest Path ☐Wide road ☐Leisure Square

29. Compared to before the pandemic, which water landscape do you prefer?

☐Fountain ☐Fall ☐Stream ☐River ☐The sea ☐Fall into water

30. In the public landscape, three kinds of sounds that you often perceive? (Multiple choice)

☐Traffic sound ☐Mechanical sound ☐Human activity sound ☐Natural sound ☐ Livestock sound ☐ Melody

Compared to before the pandemic, in the public landscape, Which voice do you prefer?

Voice preference(A Comparative Study on the Popularity of Leisure Tourism and Acoustic Preference in the Pandemic)

※Please rate your preference for the following sounds:

	Dislike very much	Less like	common	more like	Like very much
**Traffic sound**					
Farming sound	-2	-1	0	1	2
Vehicle engine noise	-2	-1	0	1	2
Road traffic noise	-2	-1	0	1	2
Bicycle bells	-2	-1	0	1	2
Ambulances sirens	-2	-1	0	1	2
Police sirens	-2	-1	0	1	2
**Mechanical sound**					
Construction noise	-2	-1	0	1	2
Handmade sound	-2	-1	0	1	2
**Human activity sound**					
Footsteps	-2	-1	0	1	2
Conversational voice	-2	-1	0	1	2
Strangers call	-2	-1	0	1	2
Children frolic	-2	-1	0	1	2
Babies crying	-2	-1	0	1	2
Busking	-2	-1	0	1	2
Dialect Hawking	-2	-1	0	1	2
Coughing	-2	-1	0	1	2
Crowd noise	-2	-1	0	1	2
Sneezing	-2	-1	0	1	2
**Natural sound**					
Rustle of leaves	-2	-1	0	1	2
Wind howling	-2	-1	0	1	2
Flowing waters	-2	-1	0	1	2
Torrential rain	-2	-1	0	1	2
Birds singing	-2	-1	0	1	2
**Livestock sound**					
Cockcrow	-2	-1	0	1	2
Livestock call	-2	-1	0	1	2
Pigs, cows and sheep noises	-2	-1	0	1	2
The chirping of cicadas	-2	-1	0	1	2
Croaking of frogs	-2	-1	0	1	2
**Melody**					
Temple bells	-2	-1	0	1	2
Pop music	-2	-1	0	1	2
Festival songs	-2	-1	0	1	2
Wedding March	-2	-1	0	1	2
Funeral music	-2	-1	0	1	2
Local folk art	-2	-1	0	1	2
Square dance music	-2	-1	0	1	2
